# Counting Footsteps with a Pedometer to Improve HMW Adiponectin and Metabolic Syndrome among Young Female Adults in the United Arab Emirates

**DOI:** 10.1155/2018/1597840

**Published:** 2018-12-06

**Authors:** Hayder Hasan, Amita Attlee, Hamid Jan Bin Jan Mohamed, Norliyana Aris, Wan Abdul Manan Bin Wan Muda

**Affiliations:** ^1^Department of Clinical Nutrition and Dietetics, College of Health Sciences, University of Sharjah, Sharjah, UAE; ^2^Diabetes and Metabolic Syndrome Research Group, Research Institute for Medical and Health Sciences, University of Sharjah, Sharjah, UAE; ^3^Nutrition and Health Department, College of Food and Agriculture, United Arab Emirates University, Al Ain, UAE; ^4^School of Health Sciences, Universiti Sains Malaysia, Kubang Kerian, Kota Bharu, Kelantan, Malaysia; ^5^Faculty of Food Science and Nutrition, Universiti Malaysia Sabah, Kota Kinabalu, Sabah, Malaysia; ^6^Center for Southeast Asia Studies, Kyoto University, Kyoto, Japan

## Abstract

**Introduction:**

Physical activity (PA) may improve cardiometabolic fitness and increase high-molecular-weight adiponectin (HMW-Adip). The pedometer is an effective, user-friendly device to monitor PA with the aim of improving health. This study examined how counting footsteps, using a pedometer, might affect HMW-Adip and MetS components among young females.

**Methods:**

Fifty-two females (21.43 ± 4.8 years) were divided into “normal” (BMI = 18–24.9 kg/m^2^) and “high” (BMI ≥ 25 kg/m^2^) BMI groups. Participants wore pedometers throughout the day for nine weeks. Pre-post intervention tests performed on anthropometric, biochemical, and nutrient intake variables were tested at *p* ≤ 0.05.

**Results:**

Participants walked 7056 ± 1570 footsteps/day without a significant difference between normal (7488.49 ± 1098) and high (6739.18 ± 1793) BMI groups. After week 9, the normal BMI group improved significantly in BMI, body fat mass (BFM), and waist-hip ratio (WHR). Additionally, percent body fat, waist circumference (WC), and visceral fat area also reduced significantly in the high BMI group. A significant decrease in triglycerides (TG) (71.62 ± 29.22 vs. 62.50 ± 29.16 mg/dl, *p*=0.003) and insulin (21.7 ± 8.33 *µ*U/l vs. 18.64 ± 8.25 *µ*U/l, *p*=0.046) and increase in HMW-Adip (3.77 ± 0.46 vs. 3.80 ± 0.44 *μ*g/ml, *p*=0.034) were recorded in the high BMI group. All participants exhibited significant inverse correlations between daily footsteps and BMI (*r*=−0.33, *p*=0.017), BFM (*r*=−0.29, *p*=0.037), WHR (*r*=−0.401, *p*=0.003), and MetS score (*r*=−0.49, *p* < 0.001) and positive correlation with HMW-Adip (*r*=0.331, *p*=0.017). A positive correlation with systolic (*r*=0.46, *p*=0.011) and diastolic (*r*=0.39, *p*=0.031) blood pressures and inverse correlation with the MetS score (*r*=−0.5, *p*=0.005) were evident in the high BMI group.

**Conclusion:**

Counting footsteps using a pedometer is effective in improving MetS components (obesity, TG) and increasing HMW-Adip levels.

## 1. Introduction

Increasing prevalence of obesity has been attributed in part to lifestyle changes and characterized by faulty dietary habits and low physical activity (PA). Studies show a significant decrease in the level of PA in everyday occupation-related and household management of PA [1]. This decline in energy expenditure substantially contributes to increased body weight in men and women, and the Global Status Report (2014) indicates that the prevalence of insufficient PA in United Arab Emirates (UAE) adults is 38.4% [2].

Physical activity counteracts the development of obesity and reduces the risk factors associated with cardiovascular disease (CVD), type 2 diabetes mellitus (T2DM), and metabolic syndrome (MetS) [3]. Moreover, PA can improve cardiorespiratory fitness and reduce the odds of death resulting from cardiovascular (CV) events, thereby decreasing the burden and hazard of obesity in societies [4]. Furthermore, PA and healthy eating patterns are important for normal-weight individuals to maintain a healthy lifestyle and to decrease morbidity and mortality rates. Hence, many researchers report the positive impact of lifestyle intervention, including diet and exercise, on multiple cardiometabolic risk factors in different age groups, sexes, and races [5]. While diet can induce weight loss, exercise can limit or deter almost all of the obesity-related cardiometabolic risks and help counteract the permissive and affluent environments that predispose persons to weight gain [6].

In order to promote and maintain health, most public health guidelines recommend accumulating at least 30 minutes per day, on most days of the week (equivalent to 150 minutes per week), of moderate-intensity physical activity (MIPA) [7]. Overweight and obese individuals should accumulate at least double this time of PA to induce favorable effects and to attain health benefits [8]. Despite being aware of the health benefits of PA, evidence suggests that many people may find it difficult to engage in structured (supervised) exercise programs as compared to lifestyle-based (home-based) PA [9]. Also, not all people find it easy to measure the intensity of their physical activities from any guidelines or instructions [10].

Walking can be a practical and enjoyable way to modify a sedentary lifestyle and promote health. Walking, especially with a pedometer, can be an effective way to increase the level of PA [11]. Pedometers are among the few widely available tools, which are effective in monitoring steps and PA, and they are accessible to the general public. The combination of having step goals with immediate feedback from a pedometer was found to be an effective means of increasing levels of PA in overweight and obese individuals [12].

It has been suggested that accumulating about 8,000 steps per day is a good surrogate for 30 minutes of daily moderate-to-vigorous PA, while accumulating 7,000 steps per day everyday of the week is consistent with obtaining 150 minutes of weekly moderate-to-vigorous PA [13]. Therefore, walking with a pedometer appears to be an easy and effective means of monitoring and increasing the level of PA, thus promoting health.

Adiponectin is the most abundant adipocytokine that is exclusively secreted by the adipocytes. Adiponectin exhibits insulin-sensitizing, fat-burning [14], antiatherogenic, and anti-inflammatory properties [15]. It is distinct from other adipokines in that the production and circulating level of adiponectin decreases with obesity. Hence, low adiponectin levels are commonly reported in obesity and related disorders such as T2DM, increased risk of CV events [16], and certain types of cancer [17]. Additionally, low adiponectin levels in healthy individuals may be linked to the increased risk of CV events [18]. Adiponectin has been reported to play a protective role in the development of MetS [19]. However, available data regarding the effects of physical exercise on serum levels of adiponectin are limited and conflicting [20]. Several studies have shown that exercise programs of six weeks to six months do not induce changes in adiponectin levels [21], while others reported that even after two to three sessions of moderate exercise, a notable elevation in serum levels of adiponectin was noticeable and persisted for more than two months without a significant change in body weight [22]. Recently, Kim et al. found that regular exercise for 24 weeks increased cardiorespiratory fitness and improved the circulating levels of high-molecular-weight adiponectin (HMW-Adip) among obese middle-aged Korean women and suggested that obesity and MetS can effectively be prevented or treated through exercise [23].

The aim of this study is to examine the effects of counting footsteps, using a pedometer, on the HMW-Adip and MetS components in normal and overweight/obese young female adults in the UAE.

## 2. Methods

### 2.1. Study Design and Subjects

In this quasi-experimental pretest-posttest study, 60 adult female participants were recruited, 52 of which completed the study. Eight participants were excluded because either they lost the pedometer (2) or they did not use the pedometer on regular daily basis (6). The participants were recruited from the University of Sharjah in the UAE and divided into two subgroups based on their body mass index: the normal BMI group (BMI=18–24.9 kg/m^2^) and high BMI group (BMI ≥ 25 kg/m^2^), indicating overweight/obese conditions. Subjects were excluded if they were underweight, pregnant, or lactating, had bariatric or recent surgery, were following dietary modifications for weight management, or had any of the diagnosed chronic diseases such as musculoskeletal disorders, heart failure, diabetes mellitus, hypertension, dyslipidemia, or cancer.

The study was approved by the Ethics and Research Committee (FWA Reg. No. 00007718; IRB Reg. No. 00004494) at the University of Sharjah and Human Research and Ethics Committee at the Universiti Sains Malaysia. Written informed consent was obtained from each participant before collecting the data.

### 2.2. Measurements

#### 2.2.1. Pedometer

Each participant wore a pedometer (KenzLifeCoder e-step; Suzuken Company, Japan) throughout the day, except during bathing and sleeping. This brand of pedometer has been found to be suitable for most research purposes [24]. The sex, height, weight, and age of each participant were manually entered into each participant's pedometer, which counted their footsteps and estimated the calories burnt. The pedometer was attached to a belt, either to the right or to the left on the waistline, for the convenience of each participant. At bedtime, the participant recorded the steps and calories accumulated during the day in a physical activity logbook. The participants were asked to follow their routine activities during the study period. The participants were requested to wear the pedometer for nine consecutive weeks in order to estimate their habitual physical activity [25].

Weekly follow-up was conducted to ensure the continuity and smooth progress of the study. Devices were checked, record logbooks were handed in, and participants were encouraged to share any doubts or queries throughout the study.

In order to measure and motivate PA, a zone-based hierarchy was adopted [26]. Accordingly, if the participant accumulated daily steps of less than 5000, 5000–7499, 7500–9999, ≥10,000–12,499, or ≥12,500, then they were considered sedentary, low active, somewhat active, active, or highly active, respectively.

#### 2.2.2. Anthropometric Assessments

Anthropometric assessments were carried out in the morning after the overnight fasting of the participants. Body height was measured to the nearest 0.1 cm, using a stadiometer (Seca 213, capacity 205 cm; Seca Company, USA). Waist circumference (WC) was also measured to the nearest 0.1 cm, using a measuring tape (Seca 201, capacity 205 cm; Seca Company, USA) at the midpoint between the lower costal margin and the iliac crest [27]. Hip circumference was measured to the nearest 0.1 cm at the level of the widest portion of the buttocks [27]. The waist-hip ratio (WHR) was determined by dividing the WC by the maximum hip circumference.

A body composition analyzer (BCA) machine (InBody 230; Biospace Company, Korea), based on the principle of bioelectrical impedance, was used to measure weight, BMI, body fat mass (BFM), percentage of body fat (PBF), visceral fat area (VFA), and fat-free mass (FFM).

The blood pressure of participants was recorded in mmHg, using a mercury sphygmomanometer (Gima, Italy) and a stethoscope (MDF777; MDF Instruments, USA).

#### 2.2.3. Biochemical Parameters

Blood samples were drawn from the participants after their overnight fasting. Within three hours of venipuncture, whole blood samples were centrifuged and separated, and serum portions were frozen at −80°C for future analysis. Biochemical parameters, such as total cholesterol (T-Chol), triglycerides (TG), high-density lipoprotein cholesterol (HDL-C), and fasting glucose levels, were analyzed using standard assay kits (Laboratories Ltd., County Antrim, UK). The low-density lipoprotein cholesterol (LDL-C) was calculated using the Friedewald formula [28].

Serum insulin levels were measured using a commercially available enzyme-linked immunosorbent assay (ELISA) kit (Immunospec Corporation, CA, USA). The sensitivity of the kits was 2.0 *µ*IU/ml. The homeostasis model assessment of insulin resistance (HOMA-IR) was calculated using the previously described formula [29].

Serum HMW-Adip levels were measured by enzyme-linked immunosorbent assay (ELISA) kits (ALPCO Diagnostics, Salem, NH, USA). The sensitivity of the kits was 0.019 ng/mL, whereas the interassay and intra-assay coefficients were 5.7% and 5.0%, respectively.

#### 2.2.4. Metabolic Score

The metabolic syndrome score (MetS score) is the summation of the number of individual components of the metabolic syndrome (i.e., if the participant had two components of MetS, the score would be 2).

#### 2.2.5. Nutrient Intake

The nutrient intake of the participants was assessed from their usual dietary intake using a valid and reliable 24-hour diet recall method [27]. The ESHA nutrient analysis software program was used (Food Processor for Windows, version 7.71; ESHA Research, Salem, OR, USA) to calculate the nutrient intake of the participants in the pre- and postintervention phases.

#### 2.2.6. Educational Material to Promote Physical Activity

The participants were advised to walk 10,000 steps per day and were provided with the educational material developed from recommendations and guidelines to promote lifestyle modification [7, 8]. The material included information on the impact of increased body weight on health and lifestyle modification (dietary and PA). A video link was shared with the participants as a guide to achieve the goal of reaching 10,000 steps per day and increasing their level of PA.

### 2.3. Statistical Analysis

Statistical analyses were performed using SPSS version 17.0 (SPSS, Chicago, IL). The variables were expressed as means and standard deviations. A paired dependent and independent Student's *t*-test was used for comparison between pre- and postintervention variables. Pearson's correlation coefficient was calculated as a measure of the relationship between different tested variables. Multiple logistic regressions were performed to indicate the best associations between tested variables and MetS components. A *p* value <0.05 was used to determine statistical significance.

## 3. Results

Fifty-two females aged 21.43 ± 4.8 years participated in this pre-post intervention study. They wore the pedometers for 61.53 ± 1.37 days (∼9 weeks) and took an average of 7056 ± 1570 steps per day.

**Figure 1:**
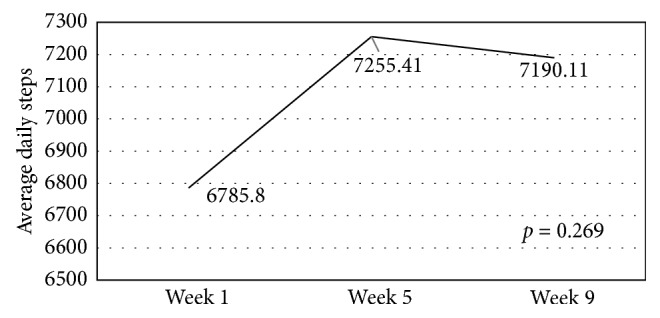
Footsteps count of participants during weeks 1, 5, and 9 (*N*=52).

There was a noticeable increase in the steps count from week 1 to week 9, being highest in week 5; however, no significant differences were demonstrated (Figure 1).

Table 1 shows changes in the anthropometric measurements (including body dimensions), body composition, blood pressure, and biochemical parameters before and after the intervention. A significant reduction in all measured parameters was observed, with the exception of FFM and blood pressure.

Conversely, no significant changes appeared in the biochemical parameters, with the exception of LDL-C and HMW-Adip. The LDL-C decreased from 63.8 ± 18.04 mg/dl before intervention to 59.17 ± 18.61 mg/dl after intervention (*p*=0.01), and the HMW-Adip increased from 4.08 ± 0.55 *μ*g/ml at baseline to 4.15 ± 0.53 *μ*g/ml at the end of the intervention (*p*=0.03) (Table 1).

Table 2 demonstrates no significant differences in the intake of macronutrients by participants before and after the intervention.

Table 3 illustrates the effects of the number of daily footsteps on anthropometric measurements, blood pressure, and biochemical parameters in participants with normal BMI (*N*=22) and high BMI (*N*=30). The normal BMI group reported a higher daily steps count compared to that of participants in the high BMI group (7488.49 ± 1098 vs. 6739.18 ± 1793), yet the difference was not statistically significant (*p*=0.089). At the end of the ninth week, however, the normal BMI group showed a significant reduction in BMI, BFM, and WHR. Conversely, the high BMI group demonstrated a significant decrease in BMI, BFM, PBF, WC, and VFA.

The normal BMI group showed no significant changes in the serum levels of the measured biochemical parameters, whereas the high BMI group showed a significant decrease in the levels of TG (71.62 ± 29.22 mg/dl vs. 62.50 ± 29.16 mg/dl, *p*=0.003) and insulin (21.7 ± 8.33 *µ*U/l vs. 18.64 ± 8.25 *µ*U/l, *p*=0.046), along with a significant increase in the serum levels.

Table 4 shows the comparison of anthropometric and biochemical parameters in participants who walked more than the average steps per day (>7056) and those who walked less than 7056 steps per day. The former group had 24 participants and walked 8243.6 ± 843 steps/day with significant improvements in only two parameters by the end of the ninth week period: WHR (0.87 ± 0.05 vs. 0.86 ± 0.05, *p*=0.046) and TG (60.5 ± 26.6 vs. 53.0 ± 23.1 mg/dl, *p*=0.03). On the contrary, those who walked <7056 steps/day (*N*=28) walked 6038.4 ± 1313 steps/day with significant changes in the BMI (29.5 ± 6.1 vs. 29.1 ± 5.9 kg/m^2^, *p*=0.003), BFM (33.2 ± 13.1 vs. 32.2 ± 4.9 kg, *p* < 0.001), PBF (42.3 ± 7.9 vs. 41.3 ± 8.2%, *p*=0.001), WC (85.8 ± 14.7 vs. 83.2 ± 12.9 cm, *p* < 0.001), VFA (109.7 ± 34.8 vs. 103.5 ± 30.5 cm^2^, *p* < 0.001), and serum level of HMW-Adip (3.93 ± 0.6 vs. 3.96 ± 0.5 *μ*g/ml, *p*=0.004).

Table 5 illustrates correlations between the daily steps count and anthropometric measurements and blood pressure after intervention. In all participants, the number of steps per day was inversely correlated with all anthropometric measurements and body composition parameters, showing a significant correlation with BMI (*r*=−0.33, *p*=0.017), BFM (*r*=−0.29, *p*=0.037), and WHR (*r*=−0.401, *p*=0.003). Furthermore, a strong, significant inverse correlation was established between the daily steps count and MetS score (*r*=−0.49, *p* < 0.001). In the normal BMI group, the number of daily steps showed no significant correlation with anthropometric measurements and blood pressure, while the high BMI group demonstrated significant positive correlations with systolic and diastolic blood pressures (*r*=0.46, *p*=0.011, and *r*=0.39, *p*=0.031, respectively) and significant inverse correlations with the MetS score (*r*=−0.5, *p*=0.005).

The number of steps did not reveal significant correlations with the biochemical parameters, with the exception of a significant positive correlation with HMW-Adip (*r*=0.331, *p*=0.017) in the 52 participants combined. Conversely, both the normal and high BMI groups showed no significant correlations between the number of steps and biochemical parameters, respectively.

Table 6 obviously shows that the trend of correlations between the serum levels of HMW adiponectin and different parameters before and after intervention was the same. However, the correlations were stronger after the intervention. The serum levels of HMW adiponectin demonstrated highly significant negative correlations with weight, BMI, BFM, FFM, PBF, WHR, WC, and VFA. On the contrary, the significant negative correlations of serum levels of HMW adiponectin with SBP and DBP at the baseline disappeared after intervention.

## 4. Discussion

Despite the lack of randomization, the quasi-experimental pretest-posttest research design of this study generated results for general trends. Such a design may be used to estimate the causal impact of an intervention on a target population. The findings of the current study focus on the level of PA among a group of young adult females in the UAE. They showed that an average of 7056 ± 1570 steps per day may result in favorable effects on anthropometric measures, the serum lipid profile, and HMW-Adip after the intervention period particularly in overweight/obese participants.

The study population accumulated an average of little more than 7,000 steps per day, suggesting that the goal of 10,000 steps per day was unrealistic for this particular study population. Although there was no significant change in the daily steps count from the start to the finish of the study, an increase in steps appeared somewhat more prominent in the middle of the study period. This may indicate that observing steps count, and receiving immediate feedback through the use of a pedometer, served to motivate the participants to achieve a higher number of steps [12]. 7,000 steps per day, nonetheless, classify the females in this study exhibiting lifestyles of low activity [26]. Similarly, a study conducted in Saudi Arabia, involving male and female teens (aged 16.1 ± 1.1 years), indicated that the mean steps count was close to 7,000 steps per day [30]. These findings are consistent with the results of a study conducted in 38 Muslim countries, where the prevalence of physical inactivity in the Muslim world was higher than that in non-Muslim countries and the variation was mainly due to higher rates among Arabs [31].

It appears that even a trivial increase in the number of steps was able to have a positive impact on obesity indices of the 52 participants. The participants with normal BMI had a marginally higher daily steps count compared to participants with high BMI; however, the latter group showed significant improvement in the anthropometric and biochemical parameters after increasing their PA. In Japan, individuals with a normal BMI were able to achieve 8,211 steps per day [21]. The study lasted for 50 days, at the end of which the participants who walked more than 8,000 steps per day showed a significant reduction in BMI, systolic blood pressure, and triglycerides. In Turkey, a four-week intervention program, including walking and dietary restriction, revealed improvement in the lipid profile [32]. Intervention in the present study was approximately nine weeks without dietary restriction and there appeared minimal, yet significant, improvement in almost all anthropometric measures. The positive biochemical changes were particularly apparent in the high BMI group, which may be attributed to a significant reduction in obesity measures and central obesity (WC and VFA), in particular. These observations are in line with those of the previous study in which the exercise in overweight and obese individuals improved anthropometric measures, such as BMI and WC [5]. In addition, walking has been proposed as an effective and safe lifestyle modification approach against central obesity [33].

Our study showed that participants with high and abnormal BMI showed a significant reduction in the serum level of TG and insulin and a significant increase in the level of HMW-Adip at the end of the ninth week. Such changes in the obese participants may have been due to a higher absolute energy expenditure in obese females compared to the lean females during PA [34] despite lower daily steps count. Researchers found that, after 12 weeks of training, there was a decrease in the TG levels, together with a drop in the insulin resistance indicator [35]. Similarly, Hong et al. indicated that exercise could positively enhance markers of insulin resistance with an increase in serum adiponectin levels in obese females [33]. The biochemical changes that occurred in the obese participants were attributed to a decrease in BFM, particularly VFA, through moderately intense exercise [36]. Furthermore, a reduction in the body weight might result in a decrease in the insulin level with a subsequent improvement in insulin resistance [37].

Moreover, the decreased level of LDL-C at the end of the study could be due to weight loss [38]. These findings correspond with the findings of an earlier study, which found that three weeks of moderately intense PA can promote favorable effects on body weight, BMI, and serum levels of total cholesterol and LDL-C [39]. However, other researchers have found changes in anthropometric measures, such as BMI and WC, after exercise without a clear effect on serum lipids [5]. The insignificant change in HDL-C levels, in the current study, may most likely be attributed to the relatively low HDL-C level before intervention that may have led to resistance to the changes after exercise [40]. Hence, exercise may increase the HDL-C level in people who have a normal or initially high level of HDL-C before starting a PA regime [41]. Another postulation is that the participants did not reach the duration and volume threshold of PA required to increase the level of HDL-C [38].

The significant inverse relationship between the daily steps count and MetS score was in agreement with a previous study [42]. In addition, the decrease in the WC improved the lipid profile, decreased insulin levels, and increased HMW-Adip levels after nine weeks of walking with a pedometer. Adding to that, the stronger inverse correlations of HMW adiponectin with obesity measures after the intervention indicate that walking with a pedometer may be an effective strategy for the prevention and treatment of MetS [43].

## 5. Conclusions

This study indicates that the participants in the “low active lifestyle” group benefitted from counting steps, using a pedometer, which improved their MetS and MetS components and reduced obesity measures while decreasing LDL-C and increasing HMW-Adip levels. The positive biochemical changes were most likely due to the decrease of the visceral fat. Therefore, the use of a pedometer may be viewed as an effective and safe approach and motivational tool to promote a healthy and active lifestyle and to combat the development of MetS.

## Figures and Tables

**Table 1:** Changes in the anthropometric measurements, blood pressure, and biochemical measurements of participants before and after intervention (*N*=52).

Variables	Before intervention (baseline)	After intervention (after 9 weeks)	*p* value
Weight (kg)	70.6 ± 15.6	69.9 ± 15.2	0.002
Body mass index (kg/m^2^)	27.5 ± 5.6	27.2 ± 5.49	0.002
Body fat mass (kg)	29.13 ± 11.9	28.08 ± 11.82	0.001
Visceral fat area (cm^2^)	98.15 ± 32.3	94.94 ± 29.3	0.007
Fat-free mass (kg)	41.7 ± 4.7	42.2 ± 5.4	0.117
Percentage of body fat (%)	39.8 ± 7.79	38.7 ± 8.23	0.011
Waist-hip ratio	0.907 ± 0.06	0.901 ± 0.06	0.025
Waist circumference (cm)	80.9 ± 13.68	79.55 ± 12.43	0.007
Systolic blood pressure (mmHg)	113.79 ± 7.24	114.4 ± 4.71	0.57
Diastolic blood pressure (mmHg)	75.38 ± 5.76	74.9 ± 2.11	0.60
Total cholesterol (mg/dl)	122.17 ± 23.21	118.69 ± 28.54	0.30
Triglycerides (mg/dl)	64.29 ± 25.0	60.48 ± 26.40	0.09
High-density lipoprotein cholesterol (mg/dl)	45.5 ± 8.22	47.43 ± 12.07	0.25
Low-density lipoprotein cholesterol (mg/dl)	63.80 ± 18.04	59.17 ± 18.61	0.01
High-molecular-weight adiponectin (*µ*g/ml)	4.08 ± 0.55	4.15 ± 0.53	0.03
Glucose (mg/dl)	72.62 ± 8.81	72.88 ± 10.84	0.85
Insulin (*µ*U/l)	20.09 ± 8.50	18.40 ± 8.47	0.24
HOMA-IR	3.64 ± 1.70	3.47 ± 2.05	0.55

**Table 2:** Changes in macronutrient intake of the participants before and after intervention (*N*=52).

Variables	Before intervention (baseline)	After intervention (after 9 weeks)	*p* value
Carbohydrates (g)	163.00 ± 56.75	150.44 ± 4.13	0.054
Fat (g)	51.35 ± 18.93	52.68 ± 22.26	0.54
Protein (g)	48.52 ± 15.72	47.63 ± 17.60	0.56
Calories (kcal)	1292.80 ± 373.89	1249.78 ± 390.08	0.33

**Table 3:** Comparisons of the means of obesity measures, blood pressure, and biochemical parameters in participants with normal and high BMIs before and after intervention (*N*=52).

	BMI = 18–24.9 kg/m^2^ (*N*=22)			BMI ≥ 25 kg/m^2^ (*N*=30)		
Average steps/day	7488.49 ± 1098			6739.18 ± 1793		
Variables	Baseline	After 9 weeks	*p* value^*∗*^	Baseline	After 9 weeks	*p* value^*∗*^
Body mass index (kg/m^2^)	22.6 ± 1.3	22.4 ± 1.4	0.002	31.1 ± 4.8	30.8 ± 4.6	0.029
Body fat mass (kg)	18.9 ± 3.5	18.3 ± 3	0.032	36.6 ± 10.2	35.3 ± 10.6	0.032
Fat-free mass (kg)	39.2 ± 3.0	39.5 ± 3.0	0.329	43.6 ± 4.97	44.16 ± 5.9	0.20
Percentage of body fat (%)	32.6 ± 4.3	32.0 ± 3.6	0.266	45.1 ± 5.1	43.6 ± 7.1	0.024
Waist-hip ratio	0.85 ± 0.0	0.84 ± 0.03	0.002	0.95 ± 0.04	0.94 ± 0.05	0.185
Waist circumference (cm)	70.8 ± 6.8	70.4 ± 5.8	0.644	88.3 ± 12.7	86.26 ± 11.7	0.001
Visceral fat area (cm^2^)	74.2 ± 16	73.3 ± 13.8	0.63	115.7 ± 30	110.7 ± 27.7	0.034
Systolic blood pressure (mmHg)	110.8 ± 6.6	112.7 ± 4.3	0.14	116 ± 7	115.66 ± 4.7	0.84
Diastolic blood pressure (mmHg)	73.2 ± 6.3	73.1 ± 4.5	0.92	77 ± 4.7	76.26 ± 4.8	0.52
Total cholesterol (mg/dl)	118.9 ± 20.9	115.6 ± 21. 8	0.50	124.5 ± 24.8	121 ± 32.8	0.45
Triglycerides (mg/dl)	54.3 ± 12.5	57.72 ± 22.5	0.28	71.6 ± 29.2	62.5 ± 29.16	0.003
High-density lipoprotein cholesterol (mg/dl)	44.4 ± 6	46.80 ± 11.1	0.16	46.30 ± 9.5	47.89 ± 12.9	0.55
Low-density lipoprotein cholesterol (mg/dl)	63.6 ± 17.4	57.23 ± 13.7	0.087	63.92 ± 18.7	60.58 ± 21.6	0.117
High-molecular-weight adiponectin (*μ*g/ml)	4.5 ± 0.3	4.62 ± 0.1	0.099	3.77 ± 0.4	3.80 ± 0.4	0.034
Glucose (mg/dl)	71.6 ± 9.4	69.77 ± 9.9	0.363	73.36 ± 8.4	75.16 ± 11.1	0.346
Insulin (*µ*U/l)	17.1 ± 8.1	17.5 ± 8.7	0.89	21.7 ± 8.3	18.64 ± 8.2	0.046
HOMA-IR	3.03 ± 1.5	3.1 ± 2.0	0.84	3.99 ± 1.7	3.60 ± 2.1	0.248

^*∗*^ *p*  value > 0.05 indicates insignificant difference.

**Table 4:** Comparisons of means of obesity measures, blood pressure, and biochemical parameters in participants who walked more than and less than average steps per day (7056 steps) before and after intervention (*N*=52).

	Participants who walked > 7056 steps/day (*N*=24)			Participants who walked < 7056 steps/day (*N*=28)		
Average steps/day	8243.6 ± 843			6038.4 ± 1313		
Variables	Baseline	After 9 weeks	*p* value^*∗*^	Baseline	After 9 weeks	*p* value^*∗*^
Body mass index (kg/m^2^)	25.1 ± 3.8	25.0 ± 4.0	0.20	29.5 ± 6.1	29.1 ± 5.9	0.003
Body fat mass (kg)	24.3 ± 8.2	23.3 ± 8.3	0.08	33.2 ± 13.1	32.2 ± 4.9	<0.001
Fat-free mass (kg)	40.7 ± 4.3	41.5 ± 6.3	0.16	42.6 ± 4.9	42.7 ± 4.5	0.47
Percentage of body fat (%)	36.9 ± 6.6	35.7 ± 7.2	0.17	42.3 ± 7.9	41.3 ± 8.2	0.001
Waist-hip ratio	0.87 ± 0.05	0.86 ± 0.05	0.046	0.93 ± 0.05	0.93 ± 0.05	0.31
Waist circumference (cm)	75.1 ± 9.8	75.3 ± 10.5	0.12	85.8 ± 14.7	83.2 ± 12.9	<0.001
Visceral fat area (cm^2^)	84.6 ± 23.2	84.7 ± 24.9	0.94	109.7 ± 34.8	103.5 ± 30.5	<0.001
Systolic blood pressure (mmHg)	114.4 ± 6.9	114.7 ± 4.7	0.79	113.2 ± 7.6	114.1 ± 4.7	0.61
Diastolic blood pressure (mmHg)	76.2 ± 4.7	75.3 ± 4.8	0.32	74.6 ± 6.5	74.6 ± 5.4	1
Total cholesterol (mg/dl)	121.3 ± 19.9	112.2 ± 24.1	0.08	122.9 ± 26.0	124.2 ± 31.2	0.76
Triglycerides (mg/dl)	60.5 ± 26.6	53.0 ± 23.1	0.03	67.5 ± 23.5	66.8 ± 27.7	0.82
High-density lipoprotein cholesterol (mg/dl)	46.0 ± 6.8	44.6 ± 11.0	0.49	45.07 ± 9.3	49.8 ± 12.5	0.07
Low-density lipoprotein cholesterol (mg/dl)	63.2 ± 17.9	57.0 ± 14.8	0.07	64.3 ± 18.4	61.0 ± 21.4	0.14
High-molecular-weight adiponectin (*µ*g/ml)	4.27 ± 0.44	4.36 ± 0.4	0.12	3.93 ± 0.6	3.96 ± 0.5	0.004
Glucose (mg/dl)	71.38 ± 7.5	71.95 ± 9.3	0.78	73.7 ± 9.7	73.6 ± 9.7	0.99
Insulin (*µ*U/l)	19.3 ± 8.8	17.8 ± 8.2	0.47	20.1 ± 8.3	18.4 ± 8.7	0.38
HOMA-IR	3.43 ± 1.7	3.25 ± 1.8	0.67	3.71 ± 1.7	3.52 ± 2.2	0.62

^*∗*^ *p*  value > 0.05 indicates insignificant difference.

**Table 5:** Correlations between the daily steps count and anthropometric measurements and blood pressure after intervention (*N*=52).

Variables	All participants (*N*=52)	BMI = 18–24.9 kg/m^2^ (*N*=22)	BMI ≥ 25 kg/m^2^ (*N*=30)
	*r*	*r*	*r*
Weight	−0.239	0.28	−0.15
Body mass index	−0.330^*∗*^	0.098	−0.29
Body fat mass	−0.290^*∗*^	0.42	−0.25
Visceral fat area	−0.211	−0.135	−0.067
Fat-free mass	−0.040	−0.09	0.106
Percentage of body fat	−0.270^*∗*^	0.41	−0.27
Waist-hip ratio	−0.401^*∗*^	−0.305	−0.35^*∗*^
Waist circumference	−0.210	−0.123	−0.068
Systolic blood pressure	0.252	0.119	0.46^*∗*^
Diastolic blood pressure	0.205	0.069	0.39^*∗*^
Metabolic syndrome score	−0.49^*∗*^	−0.39	−0.5^*∗*^
Total cholesterol	0.034	0.39	−0.04
Triglycerides	0.138	0.224	0.14
High-density lipoprotein cholesterol	0.031	0.311	−0.059
Low-density lipoprotein cholesterol	−0.007	0.30	−0.066
High-molecular-weight adiponectin	0.331^*∗*^	−0.101	0.29
Glucose	−0.034	−0.121	0.03
Insulin	−0.027	−0.136	0.10
HOMA-IR	−0.118	−0.15	0.008

^*∗*^Significant at *p* < 0.05; ^*∗∗*^significant at *p* < 0.001.

**Table 6:** Correlations between the serum levels of HMW adiponectin and anthropometric measurements and biochemical parameters before and after intervention (*N*=52).

	Before intervention (baseline)	After intervention (after 9 weeks)
Weight	−0.89^*∗∗*^	−0.96^*∗∗*^
Body mass index	−0.92^*∗∗*^	−0.99^*∗∗*^
Body fat mass	−0.91^*∗∗*^	−0.93^*∗∗*^
Fat-free mass	−0.42^*∗∗*^	−0.53^*∗∗*^
Percentage of body fat	−0.88^*∗∗*^	−0.87^*∗∗*^
Waist-hip ratio	−0.85^*∗∗*^	−0.93^*∗∗*^
Total cholesterol	−0.17	−0.06
Triglycerides	−0.25	−0.26
High-density lipoprotein cholesterol	−0.21	0.02
Low-density lipoprotein cholesterol	−0.17	−0.03
Systolic blood pressure	−0.29^*∗*^	−0.08
Diastolic blood pressure	−0.34^*∗*^	−0.09
Waist circumference	−0.77^*∗∗*^	−0.88^*∗∗*^
Visceral fat area	−0.77^*∗∗*^	−0.88^*∗∗*^
Insulin	−0.14	−0.08
Glucose	−0.01	−0.20
HOMA-IR	−0.18	−0.08

^*∗*^Significant at *p* < 0.05; ^*∗∗*^significant at *p* < 0.001.

## Data Availability

The data used to support the findings of this study are available from the corresponding author upon request.
